# Infectious Diseases and Other Health Findings in Refugees Who Arrived Through National Institute for Health, Migration and Poverty (NIHMP)-Verified Humanitarian Corridors in Italy: Changes from 2018 to 2024

**DOI:** 10.3390/healthcare14040471

**Published:** 2026-02-12

**Authors:** Lavinia Bianco, Valerio Bianco, Giovanna Laurendi, Stefania Oliva, Mariarosaria Aromatario, Aline Pizzardi, Cristiano Camponi, Christian Napoli

**Affiliations:** 1 Department of Medical Surgical Sciences and Translational Medicine, “Sapienza” University of Rome, 00189 Rome, Italy; 2 National Institute for Health, Migration and Poverty (NIHMP), 00153 Rome, Italy; 3 Department of Mathematics Guido Castelnuovo, “Sapienza” University of Rome, 00185 Rome, Italy; 4 Department of Public Health and Infectious Diseases, “Sapienza” University of Rome, 00185 Rome, Italy

**Keywords:** migration flow, humanitarian corridors, PEPs, infectious disease, health findings, National Institute for Health, Migration and Poverty (NIHMP)

## Abstract

**Introduction:** Humanitarian corridors were first established in Italy in 2015 as part of the Protected Entry Procedures (PEPs). These corridors provide a safe and legal route to Europe for migrants in need of protection, offering an alternative to perilous and illegal routes. As the first filter at entry with regard to health needs, the National Institute for Health, Migration and Poverty (NIHMP) ensures an overall individual health assessment, the primary aim of which is to identify potential infectious diseases and disorders that may require management and medical inquiry. This study aims to analyze the health data related to the humanitarian corridors that Italy has implemented and in which the NIHMP has taken part between 2018 and 2024. **Materials and Methods:** Health information and organizational records were gathered for every corridor. The analysis focused on health outcomes and on the sample’s sociodemographic characteristics. If the *p*-value was less than 0.01 it was considered statistically significant. Cramer’s V was calculated to assess the strength of each statistically significant result. **Results:** A total of 1250 refugees have been able to enter Italy thanks to the 14 NIHMP-verified humanitarian corridors. In the majority of the corridors, infectious conditions are present in less than 15% of the total number of refugees, and in only one corridor are they higher than 50%. There are statistically significant differences in nationality in the inferential analysis based on all health findings and in corridor and nationality according to the inferential analysis conducted for infectious conditions. The analysis relating to the differences by other health findings show statistically significant results in corridor, number of reports of vulnerability, and nationality. Lastly, considering the analysis done for all refugees < 18 years of age, there were statistically significant results in the number of reports of vulnerability, gender, and nationality, but there were not for any kind of health data. **Conclusions:** This study is one of the first attempts at reporting and analyzing vulnerable flags and health data concerning refugees who arrived through NIHMP-verified humanitarian corridors. Certain characteristics of our sample have few or no counterparts in the literature due to the limited availability of scientific literature on the subject. Despite this, our findings have statistical significance and scientific value, highlighting the need for further research on this subject.

## 1. Introduction

The “humanitarian corridors” were first established in 2015 as an experimental protocol signed by the Italian Federation of Evangelical Churches (FCEI), the Waldensian Table, the Italian Episcopal Conference, the Community of Sant’Egidio and the relevant Italian authorities (Ministry of the Interior and Ministry of Foreign Affairs) [1,2,3,4]. A total of 1011 refugees from Lebanon were able to safely enter Italy thanks to the first protocol, which was active between 2016 and 2017 [2]. These refugees were welcomed in 18 different Italian regions using the widespread reception model (integration of refugees in local realities) [2].

Both humanitarian corridors and other “Protected Entry Procedures” (PEPs) ensure that refugees in need of protection can enter Europe safely and legally [1]. These measures are the humanitarian answer to the escalating rise in both international migration and humanitarian needs, which are fueled by increased economic inequities, the energy crisis, socio-political uncertainty, wars, population growth, and climate change [5,6]. The PEPs mainly consist of procedures for the examination of requests for international protection by different consulates, and, if they are granted, for issuing visas [1]. As an alternative, a visa is issued right away for humanitarian reasons, but it must be reviewed once the individual has arrived in the target nation [1].

Humanitarian corridors are among the few legal and safe pathways in Europe, alongside the *resettlements*, the “evacuations” that occurred during 2018, 2019, 2022 and 2024, and pilot initiatives such as university corridors for students, health corridors for minors with cardiac disorders [7], and job corridors [4,5,8]. Before arrival, the UNHCR or the other associations involved evaluate the beneficiaries in order to discover and report possible vulnerabilities to the screening personnel inside the airport of arrival. Having a beneficiary already flagged as vulnerable allows front-line decision makers, immigration officials, and all of the other figures involved to make informed decisions concerning asylum and the migration process, in particular placement and support arrangements [9].

Whether or not a particular vulnerability is reported, once the refugees reach the target nation, a mandatory “screening” or a “health check” must be done inside the airport, as required by the national guidelines [10]. The petition for international protection must be signed at the Border Office before arrival at their final location [2,3]. Both EU and non-EU nations believe that screening migrants for diseases is beneficial, particularly if focused on infectious diseases such as TB, HIV, HBV and HCV (screening for sexually transmitted diseases is more common in non-EU countries) [11,12]. Nevertheless, non-communicable diseases (NCDs), such as mental health problems (affective and stress-related disorders), anemia, hypertension, impaired fasting glucose levels (diabetes, for example), micronutrient insufficiencies, chronic lung disease, weight issues (such as overweight or obesity) and chronic pain must also be addressed [13,14]. Similarly, an inclusive screening that enables the early diagnosis of chronic and lifestyle-related conditions should replace the current infectious diseases-focused health assessment for minors [15].

Italian procedures and laws and the role of the National Institute for Health, Migration and Poverty (NIHMP) in the rescue and initial reception phase are better explained in a previous article by the same authors [16]; briefly, the Italian initial reception phase includes a mandatory medical evaluation for signs and symptoms of clinical disorders that may require urgent healthcare assistance [10,16]. Moreover, the physician performing the medical evaluation must also look for signs of trauma and/or the results of torture, as it is necessary to correctly apply art. 17 of Italian legislative decree no. 142/2015 [16,17]. Furthermore, the NIHMP does not choose the refugees nor the countries of origin, as these choices depend on the UNHCR and on the Italian government (Ministry of Interior and Ministry of Foreign Affairs) [16,18].

The first objective of this observational, quantitative study is to share and further analyze the details of the health information related to the humanitarian corridors that Italy has implemented between 2018 and 2024 with the Istituto Nazionale per la promozione della salute delle popolazioni Migranti e per il contrasto delle malattie della Povertà (INMP)—National Institute for Health, Migration and Poverty (NIHMP): as the general results and a first analysis have already been published in a previous article by the same authors [16], this study aims to share the results of new inferential comparisons focused on health aspects. This will allow us to reduce the gap in the literature highlighted by a shortage of studies on humanitarian corridors.

## 2. Materials and Methods

Between 2018 and 2024 there were 14 NIHMP-verified humanitarian corridors, which allowed 1250 asylum seekers to enter Italy. The corridors were divided as so: 3 in 2018, 4 in 2019, 1 in 2021, 3 in 2022 and 3 in 2024.

The data collection method has already been stated in a previous article by the same authors [16], in which the same database was used for a different set of analyses: each corridor’s mandatory data reported in the paper registers was gathered and entered into an anonymous worksheet. The study was conducted in accordance with the Declaration of Helsinki, and it was approved by the INMP Ethical Competent Body (n. 31_2024). The registers were kept locked in the health management offices, and the researchers were also authorized by the Sanitary Director before being able to use them.

The data gathered, which matches to the categories on the registers, included:

- The date of the corridor;
- The sociodemographic features of the refugee(s): age, gender, nationality, marital status, education level and job/profession;
- Potential vulnerability reports by the UNHCR or other relevant organizations [9];
- The refugees’ categories: family unit, single adult or unaccompanied foreign minor (UFM);
- The specifics of the family unit: size and type;
- The region of Italy of the host;
- The presence of signs, skin manifestations, or symptoms of contagious infectious conditions;
- The presence of other data and/or health findings.

Data from all of the refugees that arrived in the NIHMP-verified corridors was included. As there were no exclusion criteria, the sample considered for this study corresponds to the entire population present in the analyzed corridors. Before beginning the analysis, all of the information present in the registries was gathered by two researchers and verified by a third. Due to the nature of the information, there were not any missing data, as the dataset was based on the mandatory sections of the registers. Therefore, there could not be any empty data, and any year that was missing had to do with the fact that there were no humanitarian corridors during that year. Any non-mandatory data that could have had missing data was, in fact, not considered in the database used.

The analysis of the sample was carried out in relation to sociodemographic characteristics, possible reporting of vulnerabilities, corridor date and health data. R software, version 4.4.0 (24 April 2024 UCRT), was used; the spreadsheet did not need to undergo further modifications or optimizations before being imported into R.

The vulnerability domains used are those recognized by the UNHCR [9], as it was UNHCR personnel that filled in that section before the flight. The domains used include the following:

- Child: Unaccompanied or separated child; child accompanied by parent/s, other family members or guardians.
- Sex, Gender, Gender Identity, Sexual Orientation: Pregnant woman or girl, or nursing mother; sole or primary carer/s (of dependent child, elderly person or person with a disability); woman at risk of sexual or gender-based violence, or adult or child experiencing family violence, exploitation or abuse; person at risk of violence due to their sexual orientation and/or gender identity (LGBTI: lesbian, gay, bisexual, transgender or intersex persons).
- Health and Welfare Concerns: Physical and mental health concern; risk of suicide; disability; elderly person; substance addiction; destitution.
- Protection Needs: Refugee and asylum-seeker; survivor of torture and trauma; survivor of sexual or gender-based violence or other violent crime; victim of trafficking in persons, stateless person.
- Other: The interviewer has an opportunity to identify vulnerability factors not captured by the previous domains [9].

For the descriptive analysis, continuous variables such as age were expressed as mean and standard deviation. Categorical variables such as nationality were summarized as the number and percentage of refugees for each category.

Inferential statistical analysis was conducted to compare the data available to us, and the Chi-square test was used. A *p*-value was considered statistically significant if less than 0.01, as a stricter *p*-value threshold guarantees that our results have more certainty towards statistical significance, as it allows to reduce the risk of Type I errors (false positive). When statistically significant results were found, Cramer’s V (measure of effect size for Chi-square independence test) was calculated to evaluate the strength of the individual correlation: if the effect size (ES) is ≤0.2 the correlation is weak, if it is 0.2 < ES ≤ 0.6 the correlation is moderate and if ES > 0.6 the correlation is strong [19].

In order to allow the analysis, some of the data with small values was merged, for example, the countries of origin of Cameroon, Chad, Congo, Egypt, Ethiopia, Myanmar, Nigeria, the Syrian Arab Republic, Somalia and Yemen have been grouped in “other” as they have fewer than 5 refugees or are not even represented in the majority of corridors. For the diagnoses of skin manifestations and/or infectious diseases, only scabies and tuberculosis (TBC) were considered separately from the rest as they are the most numerically relevant pathologies within the sample; the rest of the diseases were often reported as having zero cases in various corridors and therefore had to be merged in order to allow the analysis. The merging of subgroups that were close to zero in some corridors may have led to a loss of information, but it allowed us to use the remaining information in tests with an increased robustness.

It should be mentioned that chronic non-communicable diseases and TBC were not diagnosed at the airport; instead, they were reported in NIHMP registers because they were already listed in the refugees’ paperwork upon arrival.

In the inferential statistical analysis regarding refugees under the age of 18, the “woman at risk” vulnerability was excluded as it was present only in one female refugee, whereas the “possible or confirmed pregnancy” vulnerability was not present at all. In the comparison between the individual corridors, corridors no. 8 (25 November 2021) and no. 14 (2 September 2024) were excluded as the number of refugees on the two dates was less than 5.

In the mentioned cases with fewer than 5 refugees, it was not possible to replace the Chi-square test with Fisher’s Exact Test, as the rest of the sample was too large to apply this test and obtain reliable results.

## 3. Results

The results of each humanitarian corridor are summarized per year and include:

- Distribution of females and males;
- Mean and median age;
- Number of UFMs (unaccompanied foreign minors);
- Reports of vulnerability;
- Signs, skin manifestations, or symptoms of contagious infectious conditions;
- Other data and/or health findings.

During the years 2020 and 2023 there were not any humanitarian corridors, therefore they are absent.

### 3.1. 2018

In corridors no. I and no. II, the most relevant report of vulnerability was “possible or confirmed pregnancy” (five cases and one case, respectively), whereas corridor no. III was more eclectic and had a greater number of refugees flagged as vulnerable (97 cases) (Table 1). In corridor no. III the most common reports of vulnerability were “woman at risk” (50 cases), “child at risk” (26 cases), and “specific legal, economic, and physical protection needs” (13 cases), followed by “health problem/malnutrition/chronic disease” (four cases), and “victim of gender-based and/or sexual violence” (four cases).

Regarding signs, skin manifestations, or symptoms of contagious infectious conditions, the most common diagnoses were cough lasting more than five days (17 cases) in corridor no. I and influenza-like symptoms in corridors no. II and no. III (four and three cases, respectively).

The total number of UFMs that arrived in 2018 was 18 (Table 1).

As for the other data and/or health findings, in corridors no. I and no. III, the most frequent was the category “other” (10 cases each), while in corridor no. II the most frequent was “sight issue” (three cases).

### 3.2. 2019

The most common vulnerability reports were “health problems/malnutrition/chronic disease” (20 cases) in corridor no. IV, “detainee/held” in corridors no. V and no. VI (55 and 89 cases, respectively), and “child at risk” (11 cases) in corridor no. VII.

Regarding signs, skin manifestations, or symptoms of contagious infectious conditions, the most common diagnoses included scabies in corridors no. IV and no. V (seven and five cases, respectively) and TB in corridors no. VI and no. VII (nine and two cases, respectively).

As for the other data and/or health findings, for the first three corridors of the year the most frequent health issue was a “dermatological issue” (20, 17 and six cases, respectively), while for the last corridor the most frequent other data and/or health finding was “other” (six cases).

The total number of UFMs that arrived in 2019 was 152 (Table 2).

### 3.3. 2021

In the year 2021 there was only one humanitarian corridor (humanitarian corridor no. VIII—25 November 2021). Of the three refugees that arrived, one was female (33.3%) and two were male (66.7%), the mean age was 13.67 ± 2.9, and the median age was 12.0 (Q1 12.0 and Q3 14.5).

The vulnerability rating for all three unaccompanied foreign minors was “child at risk.” Regarding signs, skin manifestations, or symptoms of contagious infectious conditions, these were found in two unaccompanied foreign minors (66.7%), and the diagnoses were fungal infection (one case, 50.0% of potentially infected refugees) and scabies (one case, 50.0% of potentially infected refugees).

As for the other data and/or health findings, these were found in all three cases (100%), and the most frequent was “other” (two cases, 66.7%).

There were three UFMs.

### 3.4. 2022

The most common vulnerability reports were “health problems/malnutrition/chronic disease” in corridors no. IX and no. XI (10 and 14 cases, respectively), while in corridor no. X the only vulnerability reported was a “possible or confirmed pregnancy”.

The most common signs, skin manifestations, or symptoms of contagious infectious conditions found were scabies in corridors no. IX and no. XI (five and four cases, respectively), and TB in corridor no. X (two cases).

As for other data and/or health findings, corridors no. IX and no. X reported the “other” category as the most frequent (four and 10 cases, respectively), while corridor no. XI reported “headache/migraine” as the most frequent (eight cases).

During the whole year, only one UFM arrived (Table 3).

### 3.5. 2024

Each of the three corridors had a different most common vulnerability report: “child at risk” (16 cases) in corridor no. XII, “health problems/malnutrition/chronic disease” (nine cases) in corridor no. XIII, and LGBTIQ refugee (one case) in corridor no. XIV.

Regarding signs, skin manifestations, or symptoms of contagious infectious conditions, corridor no. XII registered fungal infections as the most common (five cases), corridor no. XIII scabies as the most common (six cases), and corridor no. XIV did not register any.

As for the other data and/or health findings, each of the three corridors had a different most common one: “other” (11 cases) in corridor no. XII, “pain” (eight cases) in corridor no. XIII, and “hypertension” (one case) in corridor no. XIV.

During the whole year, no UFM arrived (Table 4).

### 3.6. Graphic Representations and General Distributions

**Figure 1:**
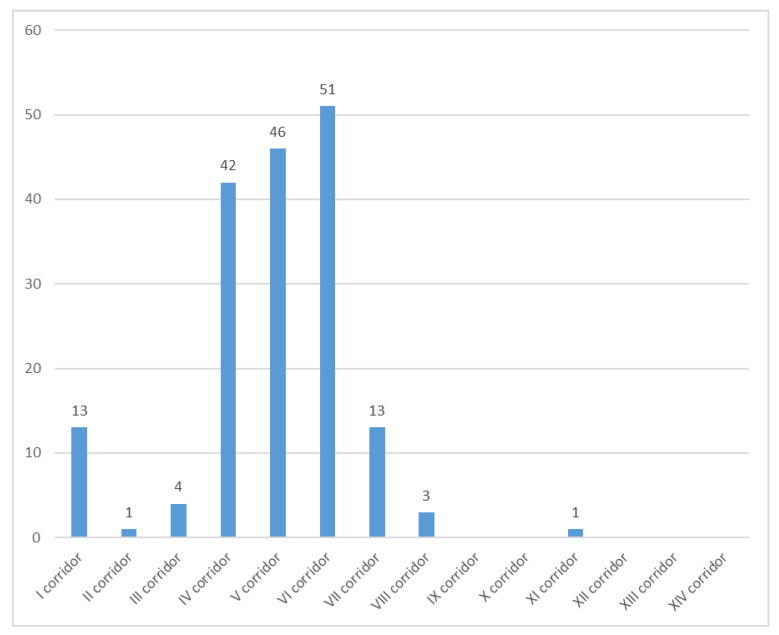
UFM (unaccompanied foreign minor) distribution in each corridor.

A graphic representation of the UFM (unaccompanied foreign minor) distribution in each corridor is shown in Figure 1.

The distribution in each corridor of vulnerability reports given to the NIHMP by the UNHCR are shown in Table 5.

The distribution in each corridor of signs, skin manifestations or symptoms of contagious infectious conditions reported by NIHMP health personnel are shown in Table 6, whereas the distribution in each corridor of the other data and/or health findings is shown in Table 7.

The results of the inferential statistical analysis of all of the health findings available are reported in Table 8 and the only significant differences are related to nationality (*p* < 0.01) and Cramer’s V shows a weak correlation (ES ≤ 0.2).

There is no statistically significant difference in relation to gender (Table 8).

The inferential analysis carried out for infectious conditions is reported in Table 9. One of the statistically significant differences is related to the individual corridors (*p* < 0.01), with a moderate correlation (0.2 < ES ≤ 0.6), and another significant *p*-value is found in relation to nationality (*p* < 0.01), with a weak correlation (ES ≤ 0.2).

There is no statistically significant difference in relation to gender (Table 9).

The results of the inferential statistical analysis by non-infectious conditions (i.e., other health findings) are reported in Table 10. Statistically significant differences are related to the individual corridors (*p* < 0.01) and to reports of vulnerability (*p* < 0.01), both with a weak correlation (ES ≤ 0.2), and to nationality (*p* < 0.01), with a moderate correlation (0.2 < ES ≤ 0.6).

There is no statistically significant difference in relation to gender (Table 10).

The inferential analysis conducted on the population under the age of eighteen based on the presence or absence of companions (parents or other relatives of age) is reported in Table 11. For the reports of vulnerability, gender and nationality, a *p*-value lower than 0.01 and a Cramer’s V between 0.2 and 0.6 are found, indicating a statistically significant difference with moderate correlation. There is no statistically significant difference in relation to infectious disease nor to other health findings (Table 11).

## 4. Discussion

Current scientific research largely concentrates on the functioning and organization of humanitarian supply networks [20,21,22,23,24], so comparing the consistency of NIHMP statistics with those currently available is difficult. Additionally, humanitarian crises are typically triggered by natural catastrophes or wars, so their rapidly evolving contexts seldom allow the collection of comprehensive and reliable quantitative information [24,25].

Asylum seekers are often said to bear a “triple burden” of infectious diseases, non-communicable conditions, and mental health issues, as their health outcomes are shaped by travel conditions, duration and method of migration, and exposure to potentially traumatic experiences [26,27,28]. Nonetheless, most Western European countries place limited emphasis on screening for non-communicable diseases upon arrival [13,29]. Instead, health assessments usually prioritize screening refugees for active tuberculosis upon arrival or soon after, regardless of TB incidence in the refugees’ country of origin, and the same strategy is applied for HIV, HBV and HCV [13,29]. As a result, the lower number of diagnoses of infectious diseases identified in our sample during the health screening upon arrival could be interpreted as a reflection of the more extensive health protections afforded to refugees arriving thanks to humanitarian corridors, both before departure and during transit. Unfortunately, a scoping review done in 2024 [30] reported that a comprehensive understanding of medicines’ access throughout the migration cycle is not currently available [30]; therefore, it is difficult to comprehend why in our sample the amount of diseases is so reduced. In fact, in eight corridors, fewer than 15% of arriving refugees are affected by diseases; in five corridors, the proportion ranges from 15% to 40%; and in only one corridor does it reach 66.7%. The lack of infectious disease diagnosis is noteworthy, as the literature shows that even if refugees and immigrants (especially women) have a higher mortality risk from infectious diseases [31] they do not constitute a significant danger to EU populations in terms of increased incidence of infectious diseases nor in triggering outbreaks [32]. Lastly, it should be mentioned that TBC was not diagnosed at the airport, as it is the UNHCR that does the screening before leaving the country of transit. Therefore, all 27 TBC-positive refugees had their status already listed in their paperwork upon arrival. Future research could explore adding a secondary screening at the airport, considering the current NIHMP provision of non-invasive tests (pregnancy test, multi-test urine strip, nasal swabs for flu and COVID-19, blood glucose test), as well as the new technological possibilities linked to rapid and non-invasive screening for TBC [33]. This screening could aid front-line decision makers to authorize immediate patient referral to the closest hospital to avoid delaying TBC-positive refugees’ care for the mandatory sanitary screening. Similarly, it could be interesting to add dried blood spot (DBS) sampling for future epidemiological research, as it showed high correlations with gold-standard venous blood sampling for various biomarkers [34,35].

With regard to chronic non-communicable diseases, the literature identifies those that are cardiovascular, musculoskeletal and respiratory as high risk in the migrant population [27]. Consequently, and given the potential additional burden they might represent on the Italian National Health Service (SSN—Servizio Sanitario Nazionale), the NIHMP collect additional information during health screening on signs, symptoms and previous diagnoses of chronic non-communicable diseases. The NIHMP acts in these settings with a dedicated methodology [16], as health service delivery to these groups can be complex, and also in consideration of the implications for both health systems and the front-line clinicians [26]. Looking at our sample, the amount of cardiovascular, musculoskeletal and respiratory diseases is very low, or even non-existent in some corridors: cardiovascular diseases were reported in a total of 30 refugees, musculoskeletal diseases were not present at all, and respiratory diseases were reported in a total of three refugees.

Psychological and/or a psychiatric issues show a particularly low prevalence in our sample, as there are only nine cases (0.7%), of which only two were minors. This finding is not confirmed by the scientific literature [36,37], including the literature which focuses on an Italian context only [38]; in fact, an article published in 2024 to assess the prevalence of psychiatric diagnoses in a few Italian reception centers showed a prevalence of 29.7% [38]. Moreover, even if psychological/psychiatric issues have higher prevalence rates in UFMs compared to accompanied foreign minors and European-born minors [39,40], our sample shows even lower rates than those reported in 2025 by Mattelin et al. [41].

It should be noted that the low incidence of both infectious and non-communicable diseases may reflect health influences during the pre-migration, transit and post-migration phases [27]. Therefore, it could be speculated that the system specific to humanitarian corridors may contribute to the different distribution of health conditions in our sample. Additionally, the reported low sensitivity of airport medical screenings could play a part [42,43]. However, this hypothesis cannot be currently tested with the available data, and further research is needed on the subject to explore these potential explanations.

Even considering the selection bias, the statistically significant differences observed with respect to nationality across all possible combinations of infectious conditions and health findings are particularly noteworthy. Notably, Sudanese individuals appear to be less prone to developing infectious conditions compared to other national groups. This finding suggests possible differences in prior exposure, immunity, or other underlying protective factors. On one hand, this result contrasts sharply with the existing scientific literature, which consistently reports that Sudan is frequently and recurrently affected by epidemic-prone diseases [44,45]; on the other hand, significant sample size differences between our study and those present in the literature (333 refugees from Sudan in our study versus 62 Sudanese refugees [44] and a non-disclosed number of Sudanese refugees [45] in the existing literature) prevent direct one-to-one comparisons.

Conversely, Eritreans seem less likely to develop non-infectious health issues. This observation may be due to distinct health profiles or background characteristics specific to the study sample, as it is not supported by the existing literature. In fact, the available data show an increasing impact of non-communicable diseases in Eritrea, such as chronic cardiovascular disease, diabetes, cancer and chronic obstructive pulmonary diseases [46]. However, the review by Abdu et al. [46] included articles that give details of results on at least 100,000 males and 100,000 females, while our sample includes 560 Eritrean refugees.

Regarding the reports of vulnerability, which are present in 451 refugees, their usefulness is undeniable. Pre-identifying beneficiaries allows front-line decision makers to set priorities and decide the order of the mandatory sanitary screening upon arrival at the airport. For instance, knowing that a refugee has diabetes ensures that she is examined by a doctor before other refugees without identified vulnerabilities. Similarly, knowing a refugee child’s medical needs enables the medical personnel to prepare the necessary equipment and expertise in advance, while information on whether the child is accompanied by an adult or is alone helps select the right accommodation. To facilitate this prioritization, the UNHCR uses a screening process that identifies the vulnerabilities and classifies the beneficiaries in low, medium or high levels of vulnerability [9]. Moreover, a refugee flagged with one or more vulnerabilities could present other non-disclosed issues, such as decreased odds of having received a COVID-19 vaccine (especially in people originating from Ménaka) or higher risk of COVID-19 exposure and infection (especially in displaced people in urban areas) [47].

However, it should be noted that vulnerability can sometimes be turned into a divisive and exclusionary tool [48], and therefore we do not wish to imply that it is the best way to establish priority upon refugees’ arrival, nor that other methods currently in use are less valid. Furthermore, even though we used the vulnerability domains identified by the UNHCR [9], it was not possible to find comparable data in the literature; this might be because even if the notion of vulnerability is often applied in both legal and policy contexts it lacks any normative foundation or universally agreed definition [48]. In any case, our findings highlighted a correlation between the reports of vulnerability and the health outcomes, including infectious conditions. In particular, in the vulnerability group “other”, it was more likely to find positive reports of health outcomes. This could hypothetically indicate that in our sample a positive health finding cannot be connected to just one specific vulnerability domain, but only to a heterogeneous group. This pattern suggests that multiple, possibly overlapping, vulnerability factors may contribute to the health profiles observed in this population. Such a suggestion could be considered substantiated by the fact that the potential factors of delayed or missed care are multifactorial [49]; socially vulnerable groups are also more likely to experience poor health outcomes and a disproportionate burden of illnesses [50].

Looking at UFMs (unaccompanied foreign minors), it is evident that in our sample the greatest number of UFMs arrived in the year 2019 (152 UFMs of the 174 total). Nevertheless, it has been reported that, at an EU level, the greater number of unaccompanied minor first-time asylum applicants among the total number of first-time asylum applicants was registered in the year 2015 (24.5% recorded), with a minimum value of 7.5% in 2019 [51]. Similarly, the increase in the unaccompanied minor first-time asylum applicants registered in 2021 and 2022 [51,52] is not reflected in our sample. However, this discrepancy could be partly attributable to the fact that the available sources cover the entire European Union rather than Italy specifically, and only a limited number of unaccompanied minors applied in Italy [52].

This study has several limitations. First, considering the limited scientific literature on this topic, it is challenging to assess the congruence of NIHMP data with those presently available; consequently, our findings are open to multiple interpretations and hypotheses rather than a single, definitive and univocal conclusion. Second, as the reported data and the medical documentation were taken from anonymous databases, it was not possible to recover additional data directly from the refugees, nor to include biomarker or laboratory-confirmed data in regard to the reported diseases. Furthermore, our analysis could not consider nor exclude potential confounding factors such as corridor composition and pre-departure health screening, and by merging some subgroups before the statistical analysis we lost some information in order to increase our tests’ robustness.

Finally, our analysis included only humanitarian corridors to Italy managed by the NIHMP, excluding any other corridors that may have happened in the same period. This inevitably created a selection bias, as corridors managed by an association other than the UNHCR and the Italian government could have involved other countries of origin and destinations, as well as varying proportions of both vulnerable refugees and UFMs.

## 5. Conclusions

The data presented and analyzed in this paper represent one of the first attempts to provide a comprehensive and extensive review of the health status of the refugees arriving in Italy though humanitarian corridors.

The limited availability of scientific articles specifically addressing humanitarian corridors suggests a significant gap in the research, especially when compared to the broader focus on humanitarian supply chains. As a result, there are characteristics unique to our sample, such as reports of vulnerability, that have applicability and statistical significance solely within our internal analysis, as comparable data is not present in the existing literature. Nevertheless, our findings indicate that data on infectious diseases is largely consistent with the existing literature, whereas results concerning non-communicable diseases diverge from it.

Overall, it is possible to conclude that our findings underscore the need for more comprehensive health assessments and the development of tailored support strategies for this population.

## Figures and Tables

**Table 1:** Result of each corridor organized in the year 2018.

	No. I—14 February	No. II—14 November	No. III—19 December
Total number of refugees	148	50	103
Females, *n* (%)	85 (57.4%)	36 (72.0%)	71 (68.9%)
Males, *n* (%)	63 (42.6%)	14 (28.0%)	32 (31.1%)
Mean age, mean ± DS	23.70 ± 9.26	20.3 ± 11.8	18.89 ± 11.9
Median age, median (Q1–Q3)	24.0 (Q1 19.0–Q3 28.25)	21.0 (Q1 11.25–Q3 28.0)	20.0 (Q1 12.0–Q3 24.0)
UFMs, *n* (%)	13 (8.8%)	1 (2.0%)	4 (3.9%)
Reports of vulnerability			
Present, *n* (%)	6 (4.1%)	1 (2.0%)	97 (94.2%)
None, *n* (%)	142 (95.9%)	49 (98.0%)	6 (5.8%)
Signs, skin manifestations, or symptoms of contagious infectious conditions			
Present, *n* (%)	55 (37.2%)	6 (12.0%)	13 (12.6%)
None, *n* (%)	93 (62.8%)	44 (88.0%)	90 (87.4%)
Other data and/or health findings			
Present, *n* (%)	43 (29.1%)	15 (30.0%)	30 (29.1%)
None, *n* (%)	105 (70.9%)	35 (70.0%)	73 (70.9%)

**Table 2:** Result of each corridor organized in the year 2019.

	No. IV—29 April	No. V—30 May	No. VI—12 September	No. VII—5 November
Total number of refugees	144	148	98	54
Females, *n* (%)	57 (39.6%)	69 (46.6%)	18 (18.4%)	29 (53.7%)
Males, *n* (%)	87 (60.4%)	79 (53.4%)	80 (81.6%)	25 (46.3%)
Mean age, mean ± DS	18.6 ± 8.3	18.34 ± 7.8	18.76 ± 5.6	19.31 ± 8.9
Median age, median (Q1–Q3)	19.0 (Q1 15.75–Q3 23.25)	19.0 (Q1 15.0-Q3 23.0)	16.0 (Q1 16.0–Q3 22.0)	20.0 (Q1 16.0–Q3 25.25)
UFMs, *n* (%)	42 (29.2%)	46 (31.1%)	51 (52.0%)	13 (24.1%)
Reports of vulnerability				
Present, *n* (%)	38 (26.4%)	85 (57.4%)	94 (95.9%)	38 (70.4%)
None, *n* (%)	106 (73.6%)	63 (42.6%)	4 (4.1%)	16 (29.6%)
Signs, skin manifestations, or symptoms of contagious infectious conditions				
Present, *n* (%)	25 (17.4%)	18 (12.2%)	32 (32.7%)	11 (20.4%)
None, *n* (%)	119 (82.6%)	130 (87.8%)	66 (67.3%)	43 (79.6%)
Other data and/or health findings				
Present, *n* (%)	65 (45.1%)	49 (33.1%)	28 (28.6%)	21 (38.9%)
None, *n* (%)	79 (54.9%)	99 (66.9%)	70 (71.4%)	33 (61.1%)

**Table 3:** Result of each corridor organized in the year 2022.

	No. IX—28 February	No. X—26 July	No. XI—30 November
Total number of refugees	97	79	101
Females, *n* (%)	32 (33.0%)	13 (16.4%)	21 (20.8%)
Males, *n* (%)	65 (67.0%)	66 (83.6%)	80 (79.2%)
Mean age, mean ± DS	25.8 ± 10.5	23.65 ± 8.9	25.36 ± 9.6
Median age, median (Q1–Q3)	24.0 (Q1 21.0–Q3 30.0)	19.0 (Q1 18.0–Q3 28.0)	23.0 (Q1 20.0–Q3 29.0)
UFMs, *n* (%)	0 (0.0%)	0 (0.0%)	1 (0.9%)
Reports of vulnerability			
Present, *n* (%)	19 (19.6%)	1 (1.3%)	17 (16.8%)
None, *n* (%)	78 (80.4%)	78 (98.7%)	84 (83.3%)
Signs, skin manifestations, or symptoms of contagious infectious conditions			
Present, *n* (%)	9 (9.3%)	7 (8.9%)	10 (9.9%)
None, *n* (%)	88 (90.7%)	72 (91.1%)	91 (90.1%)
Other data and/or health findings			
Present, *n* (%)	12 (12.4%)	30 (38.0%)	36 (35.6%)
None, *n* (%)	85 (87.6%)	49 (62.0%)	65 (64.4%)

**Table 4:** Result of each corridor organized in the year 2024.

	No. XII—7 May	No. XIII—29 July	No. XIV—2 September
Total number of refugees	119	102	4
Females, *n* (%)	53 (44.5%)	19 (18.6%)	3 (75.0%)
Males, *n* (%)	66 (55.5%)	83 (81.4%)	1 (25.0%)
Mean age, mean ± DS	23.6 ± 10.4	24.9 ± 7.2	17.0 ± 16.8
Median age, median (Q1–Q3)	23.0 (Q1 21.0–Q3 30.0)	25.0 (Q1 21.3–Q3 29.0)	17.0 (Q1 3.25–Q3 30.75)
UFMs, *n* (%)	0 (0.0%)	0 (0.0%)	0 (0.0%)
Reports of vulnerability			
Present, *n* (%)	39 (32.8%)	12 (11.8%)	3 (75.0%)
None, *n* (%)	80 (67.2%)	90 (90.2%)	1 (25.0%)
Signs, skin manifestations, or symptoms of contagious infectious conditions			
Present, *n* (%)	13 (10.9%)	22 (21.6%)	0 (0.0%)
None, *n* (%)	106 (89.1%)	80 (78.4%)	4 (100.0%)
Other data and/or health findings			
Present, *n* (%)	45 (37.8%)	36 (35.3%)	1 (25.0%)
None, *n* (%)	74 (62.2%)	66 (64.7%)	3 (75.0%)

**Table 5:** Distribution in each corridor of vulnerability reports given to the NIHMP by the UNHCR.

	I Corridor	II Corridor	III Corridor	IV Corridor	V Corridor	VI Corridor	VII Corridor	VIII Corridor	IX Corridor	X Corridor	XI Corridor	XII Corridor	XIII Corridor	XIV Corridor
Child at risk	0	0	26	0	2	2	11	3	2	0	0	16	0	0
Detainee/held	0	0	0	0	55	89	6	0	0	0	0	0	0	0
Detainee/held—victim of torture and/or physical and/or sexual violence	0	0	0	0	11	1	0	0	0	0	0	0	0	0
Possible or confirmed pregnancy	5	1	0	10	1	1	1	0	0	1	1	1	0	0
LGBTIQ	0	0	0	0	1	0	0	0	0	0	1	1	1	1
Health problems/malnutrition/chronic disease	1	0	4	20	7	1	0	0	10	0	14	13	9	0
Specific legal, economic and physical protection needs	0	0	13	0	3	0	4	0	0	0	0	0	0	0
Torture/physical violence	0	0	0	7	4	0	2	0	0	0	1	4	2	0
Victim of gender-based and/or sexual violence	0	0	4	1	1	0	7	0	0	0	0	4	0	0
Woman at risk	0	0	50	0	0	0	7	0	7	0	0	0	0	0
No report of vulnerability	142	49	6	106	63	4	16	0	78	78	84	80	90	3

**Table 6:** Distribution in each corridor of signs, skin manifestations or symptoms of contagious infectious conditions reported by NIHMP health personnel.

	I Corridor	II Corridor	III Corridor	IV Corridor	V Corridor	VI Corridor	VII Corridor	VIII Corridor	IX Corridor	X Corridor	XI Corridor	XII Corridor	XIII Corridor	XIV Corridor
Acariasis	1	0	1	0	1	0	0	0	0	0	0	0	0	0
Dysentery	1	0	0	0	0	1	0	0	0	0	0	0	0	0
Viral hepatitis	0	0	1	0	0	0	1	0	0	0	2	1	3	0
HIV+	0	0	0	0	0	0	0	0	1	0	0	0	0	0
Fungal infection	5	1	2	1	1	1	2	1	0	1	1	5	4	0
Suspected or confirmed upper respiratory tract infection	2	0	0	0	0	1	0	0	0	0	0	0	0	0
Viral infection	0	0	0	0	0	0	0	0	2	0	0	1	0	0
Hansen disease	0	0	0	0	0	0	0	0	0	0	0	0	1	0
Multiple contagious infectious conditions	0	0	0	0	2	2	0	0	0	0	0	0	0	0
Intestinal parasitosis	0	0	0	0	0	0	0	0	1	0	0	0	0	0
Pneumonia	0	0	1	0	0	0	0	0	0	0	0	0	0	0
Possible STD	1	0	1	0	0	1	0	0	0	0	0	1	0	0
Scabies	14	0	2	7	5	8	2	1	5	3	4	1	6	0
Acute nonspecific symptoms	10	0	1	2	2	3	1	0	0	0	1	2	4	0
Parainfluenza symptoms	3	4	3	6	3	2	2	0	0	0	0	1	0	0
Suspected exanthematous disease	0	1	1	0	1	0	0	0	0	0	0	0	0	0
TBC	1	0	0	5	2	9	2	0	0	2	2	1	3	0
Cough lasting more than 5 days	17	0	0	4	1	4	1	0	0	1	0	0	1	0
No signs, skin manifestations or symptoms of contagious infectious conditions	93	44	90	119	130	66	43	1	88	72	91	106	80	4

**Table 7:** Distribution in each corridor of other data and/or health findings.

	I Corridor	II Corridor	III Corridor	IV Corridor	V Corridor	VI Corridor	VII Corridor	VIII Corridor	IX Corridor	X Corridor	XI Corridor	XII Corridor	XIII Corridor	XIV Corridor
Anemia	0	0	0	0	0	0	0	0	0	1	0	0	0	0
Cardiac rhythm abnormalities	0	1	0	1	0	2	0	0	1	0	0	2	1	0
Asthma	0	0	0	0	0	0	0	0	0	0	0	3	0	0
Heart disease	0	1	0	1	0	1	1	0	0	0	0	0	0	0
Headache/migraine	6	3	3	6	4	1	0	0	1	5	8	3	7	0
Diabetes	1	0	1	0	1	0	0	0	2	0	3	1	1	0
Sight issue	1	3	1	1	1	3	1	0	0	2	3	4	3	0
Dermatological issue	9	0	2	20	17	6	1	0	0	3	4	4	7	0
Pain	8	1	5	9	10	6	4	0	0	3	3	7	8	0
Epilepsy	0	0	0	0	0	0	1	0	0	0	0	0	0	0
Pregnancy/puerperium	6	1	2	11	5	2	3	0	1	1	1	0	0	0
Hypertension	0	1	2	2	0	1	1	0	1	1	2	5	1	1
Psychological/psychiatric problems	1	1	0	0	0	1	3	1	1	1	0	0	0	0
Trauma	1	0	4	2	1	2	0	0	1	3	5	5	2	0
Other	10	3	10	12	10	3	6	2	4	10	7	11	6	0
None	105	35	73	79	99	70	33	0	85	49	65	74	66	3

**Table 8:** Results of inferential statistical analysis of all health findings (infectious and other).

	All	Presence of Both Contagious Infectious Conditions and Other Health Findings	Presence of Contagious Infectious Conditions Only	Presence of Other Health Findings Only	Absence of Both Contagious Infectious Conditions and Other Health Findings	*p*-Value *	Cramer’s V
*n*	1250	53	170	361	666	-	-
Age (years), mean (SD, min–max)	21.7 ± 9.21 (0–67)	25.4 ± 12.24 (0–67)	19.8 ± 10.1 (0–48)	23.3 ± 9.7 (0–67)	21.0 ± 9.1 (0–59)	-	-
Gender						0.568439	-
Male, *n* (%)	743 (59.4%)	33 (62.3%)	108 (63.5%)	207 (57.3%)	395 (59.3%)		
Female, *n* (%)	507 (40.6%)	20 (37.7%)	62 (36.5%)	154 (42.7%)	271 (40.7%)		
Nationality						**0.002086**	0.090969
Eritrean, *n* (%)	560 (44.8%)	24 (45.3%)	89 (52.4%)	136 (37.7%)	311 (46.7%)		
Sudanese, *n* (%)	333 (26.6%)	8 (15.1%)	32 (18.8%)	115 (31.8%)	178 (26.7%)		
Other, *n* (%)	357 (28.6%)	21 (39.6%)	49 (28.8%)	110 (30.5%)	177 (26.6%)		

* Chi-square test. **Bold:** *p*-value less than 0.01.

**Table 9:** Results of the inferential statistical analysis of the presence of infectious conditions.

	All	Absence of Contagious Infectious Conditions	Presence of Contagious Infectious Conditions	*p*-Value *	Cramer’s V
*n*	1250	1027	223	**-**	**-**
Age (years), mean (SD, min–max)	21.7 ± 9.21 (0–67)	21.8 ± 9.34 (0–67)	21.1 ± 10.8 (0–67)	**-**	**-**
Humanitarian corridors ^@^				**<0.000001**	*0.24978*
I corridor, *n* (%)	148 (11.8%)	93 (9.0%)	55 (24.8%)		
II corridor, *n* (%)	50 (4.0%)	44 (4.3%)	6 (2.8%)		
III corridor, *n* (%)	103 (8.3%)	90 (8.7%)	13 (5.8%)		
IV corridor, *n* (%)	144 (11.6%)	119 (11.6%)	25 (11.2%)		
V corridor, *n* (%)	148 (11.8%)	130 (12.6%)	18 (8.1%)		
VI corridor, *n* (%)	98 (7.8%)	66 (6.4%)	32 (14.3%)		
VII corridor, *n* (%)	54 (4.3%)	43 (4.2%)	11 (5.0%)		
IX corridor, *n* (%)	97 (7.8%)	88 (8.6%)	9 (4.0%)		
X corridor, *n* (%)	79 (6.3%)	72 (7.0%)	7 (3.1%)		
XI corridor, *n* (%)	101 (8.0%)	91 (8.9%)	10 (4.5%)		
XII corridor, *n* (%)	119 (9.5%)	106 (10.3%)	13 (5.8%)		
XIII corridor, *n* (%)	102 (8.2%)	80 (7.8%)	22 (10.0%)		
Reports of vulnerability				0.08665	-
None, *n* (%)	799 (63.9%)	664 (64.6%)	135 (60.5%)		
Woman at risk, *n* (%)	64 (5.1%)	58 (5.7%)	6 (2.8%)		
Child at risk, *n* (%)	62 (5.0%)	49 (4.8%)	13 (5.8%)		
Other, *n* (%)	325 (26.0%)	256 (24.9%)	69 (30.9%)		
Gender				0.23169	-
Male, *n* (%)	743 (59.4%)	602 (58.6%)	141 (63.2%)		
Female, *n* (%)	507 (40.6%)	425 (41.4%)	82 (36.8%)		
Nationality				**0.00507**	0.09194
Eritrean, *n* (%)	560 (44.8%)	447 (43.5%)	113 (50.7%)		
Sudanese, *n* (%)	333 (26.6%)	293 (28.6%)	40 (17.9%)		
Other, *n* (%)	357 (28.6%)	287 (27.9%)	70 (31.4%)		

* Chi-square test. @ Excluded corridors *n*. VIII (25 November 2021) and *n*. XIV (2 September 2024): numerosity less than 5, with a total of 7 refugees (0.6% of the sample) whose nationalities are in the category “other”. **Bold:** *p*-value less than 0.01. *Italics*: Cramer’s V is 0.2 < ES ≤ 0.6 (the correlation is moderate).

**Table 10:** Results of the inferential statistical analysis of other health findings (not infectious).

	All	Absence of Other Health Findings	Presence of Other Health Findings	*p*-Value *	Cramer’s V
*n*	1250	836	414	**-**	**-**
Age (years), mean (SD, min–max)	21.7 ± 9.21 (0–67)	20.7 ± 9.29 (0–59)	23.6 ± 10.03 (0–67)	**-**	**-**
Humanitarian corridors ^@^				**0.000285**	0.166914
I corridor, *n* (%)	148 (11.8%)	105 (12.6%)	43 (10.4%)		
II corridor, *n* (%)	50 (4.0%)	35 (4.2%)	15 (3.6%)		
III corridor, *n* (%)	103 (8.3%)	73 (8.7%)	30 (7.2%)		
IV corridor, *n* (%)	144 (11.6%)	79 (9.4%)	65 (15.7%)		
V corridor, *n* (%)	148 (11.8%)	99 (11.8%)	49 (11.8%)		
VI corridor, *n* (%)	98 (7.8%)	70 (8.4%)	28 (6.8%)		
VII corridor, *n* (%)	54 (4.3%)	33 (3.9%)	21 (5.1%)		
IX corridor, *n* (%)	97 (7.8%)	85 (10.2%)	12 (2.9%)		
X corridor, *n* (%)	79 (6.3%)	49 (5.9%)	30 (7.2%)		
XI corridor, *n* (%)	101 (8.0%)	65 (7.8%)	36 (8.7%)		
XII corridor, *n* (%)	119 (9.5%)	74 (8.9%)	45 (10.9%)		
XIII corridor, *n* (%)	102 (8.2%)	66 (7.9%)	36 (8.7%)		
Reports of vulnerability				**0.002371**	0.10745
None, *n* (%)	799 (63.9%)	555 (66.4%)	244 (58.9%)		
Woman at risk, *n* (%)	64 (5.1%)	46 (5.5%)	18 (4.3%)		
Child at risk, *n* (%)	62 (5.0%)	45 (5.4%)	17 (4.2%)		
Other, *n* (%)	325 (26.0%)	190 (22.7%)	135 (32.6%)		
Gender				0.494506	-
Male, *n* (%)	743 (59.4%)	503 (60.2%)	240 (57.9%)		
Female, *n* (%)	507 (40.6%)	333 (39.8%)	174 (42.1%)		
Nationality				**0.008737**	0.08708
Eritrean, *n* (%)	560 (44.8%)	400 (47.8%)	160 (38.6%)		
Sudanese, *n* (%)	333 (26.6%)	210 (25.2%)	123 (29.7%)		
Other, *n* (%)	357 (28.6%)	226 (27.0%)	131 (31.7%)		

* Chi-square test. @ Excluded corridors *n*. VIII (25 November 2021) and *n*. XIV (2 September 2024): numerosity less than 5, with a total of 7 refugees (0.6% of the sample), whose nationalities are in the category “other”. **Bold:** *p*-value less than 0.01.

**Table 11:** Results of inferential statistical analysis in members of the population <18 years old.

	All	UFM	Accompanied	*p*-Value *	Cramer’s V
*n*	326	174	152	**-**	**-**
Age (years), mean (SD, min–max)	10.6 ± 6.60 (0–17)	15.7 ± 1.07 (12–17)	4.7 ± 5.21 (0–17)	**-**	**-**
Reports of vulnerability				**<0.000001**	*0.32810*
None, *n* (%)	176 (54.0%)	85 (48.9%)	91 (59.9%)		
Child at risk, *n* (%)	62 (19.0%)	20 (11.4%)	42 (27.6%)		
Other, *n* (%)	88 (27.0%)	69 (39.7%)	19 (12.5%)		
Gender				**<0.000001**	*0.34155*
Male, *n* (%)	218 (66.9%)	143 (82.2%)	75 (49.3%)		
Female, *n* (%)	108 (33.1%)	31 (17.8%)	77 (50.7%)		
Nationality				**<0.000001**	*0.36215*
Eritrean, *n* (%)	159 (48.8%)	98 (56.3%)	61 (40.1%)		
Sudanese, *n* (%)	67 (20.5%)	12 (6.9%)	55 (36.2%)		
Other, *n* (%)	100 (30.7%)	64 (36.8%)	36 (23.7%)		
Signs, skin manifestations or symptoms of contagious infectious conditions				0.80355	-
None, *n* (%)	254 (77.9%)	137 (78.7%)	117 (77.0%)		
Present, *n* (%)	72 (22.1%)	37 (21.3%)	35 (23.0%)		
Other health findings				0.04627	-
None, *n* (%)	237 (72.7%)	118 (67.8%)	119 (78.3%)		
Present, *n* (%)	89 (27.3%)	56 (32.2%)	33 (21.7%)		

* Chi-square test. **Bold:** *p*-value less than 0.01. *Italics*: Cramer’s V is 0.2 < ES ≤ 0.6 (the correlation is moderate).

## Data Availability

The raw data supporting the conclusions of this article will be made available by the authors on request.

## Abbreviations

The following abbreviations are used in this manuscript:

EU	European Union
ES	Effect Size
FCEI	Italian Federation of Evangelic Churches
HBV	Hepatitis B Virus
HCV	Hepatitis C Virus
HIV	Human Immunodeficiency Virus
INMP	Istituto Nazionale per la promozione della salute delle popolazioni Migranti e per il contrasto delle malattie della Povertà
NCDs	Non-Communicable Diseases
NIHMP	National Institute for Health, Migration and Poverty
PEPs	Protected Entry Procedures
SSN	Servizio Sanitario Nazionale
TB	Tuberculosis
UFM	Unaccompanied Foreign Minor
UNHCR	United Nations High Commissioner for Refugees
